# Exploring the Physiological Role of Transthyretin in Glucose Metabolism in the Liver

**DOI:** 10.3390/ijms22116073

**Published:** 2021-06-04

**Authors:** Mobina Alemi, Ângela Oliveira, Sofia C. Tavares, José Ricardo Vieira, Marco G. Alves, Pedro F. Oliveira, Isabel Cardoso

**Affiliations:** 1 i3S—Instituto de Investigação e Inovação em Saúde, Universidade do Porto, 4200-135 Porto, Portugal; mobinaalemi@med.up.pt (M.A.); angela.d.a.oliveira@gmail.com (Â.O.); sofiatavares1@hotmail.com (S.C.T.); jrcruzvieira@gmail.com (J.R.V.); 2 Molecular Neurobiology, IBMC—Instituto de Biologia Molecular e Celular, Universidade do Porto, 4200-135 Porto, Portugal; 3 Faculdade de Medicina, Universidade do Porto, 4200-319 Porto, Portugal; 4 Unit for Multidisciplinary Research in Biomedicine (UMIB), Department of Anatomy, Institute of Biomedical Sciences Abel Salazar (ICBAS), University of Porto, 4050-313 Porto, Portugal; alvesmarc@gmail.com; 5 QOPNA & LAQV, Department of Chemistry, University of Aveiro, 3810-193 Aveiro, Portugal; pfobox@gmail.com; 6 Instituto de Ciências Biomédicas Abel Salazar (ICBAS), 4050-013 Porto, Portugal

**Keywords:** transthyretin, glucose metabolism, glucose transporters, liver, mitochondria

## Abstract

Transthyretin (TTR), a 55 kDa evolutionarily conserved protein, presents altered levels in several conditions, including malnutrition, inflammation, diabetes, and Alzheimer’s Disease. It has been shown that TTR is involved in several functions, such as insulin release from pancreatic β-cells, recovery of blood glucose and glucagon levels of the islets of Langerhans, food intake, and body weight. Here, the role of TTR in hepatic glucose metabolism was explored by studying the levels of glucose in mice with different TTR genetic backgrounds, namely with two copies of the *TTR* gene, TTR+/+; with only one copy, TTR+/−; and without TTR, TTR−/−. Results showed that TTR haploinsufficiency (TTR+/−) leads to higher glucose in both plasma and in primary hepatocyte culture media and lower expression of the influx glucose transporters, GLUT1, GLUT3, and GLUT4. Further, we showed that TTR haploinsufficiency decreases pyruvate kinase M type (PKM) levels in mice livers, by qRT-PCR, but it does not affect the hepatic production of the studied metabolites, as determined by 1H NMR. Finally, we demonstrated that TTR increases mitochondrial density in HepG2 cells and that TTR insufficiency triggers a higher degree of oxidative phosphorylation in the liver. Altogether, these results indicate that TTR contributes to the homeostasis of glucose by regulating the levels of glucose transporters and PKM enzyme and by protecting against mitochondrial oxidative stress.

## 1. Introduction

The liver, one of the largest organs in the human body, is able to eliminate most of the threats exposed by environmental contaminants, pharmaceuticals, and microorganisms [1] and store and produce energy, proteins, and lipids [2]. Glucose is the most important source of energy in all organisms, also produced, stored, and distributed by the liver. Plasma glucose can be transported across the hepatocyte membrane through glucose transporters (GLUTs), stored as glycogen or consumed by glycolysis, followed by tricarboxylic acid (TCA) cycle and mitochondrial oxidative phosphorylation, producing energy in the form of ATP. During mitochondrial electron transport, electrons can induce production of superoxide anions and reactive oxygen species (ROS) [3]. High concentration of ROS, occurring in several conditions [4,5,6,7,8], causes damage to proteins, lipids, and nucleic acids, further exacerbating the disease development [9].

Hepatic glucose uptake and efflux are influenced by different mechanisms, namely glycolysis, gluconeogenesis, and expression of GLUTs. Importantly, insulin resistance, hyperlipidaemia, alcohol consumption, viral infection, carcinogenesis, and many other conditions can dysregulate these systems [10]. Among liver GLUTs, GLUT2 is the major facilitative glucose transporter, and due to its low affinity and high capacity, it promotes bidirectional fluxes of glucose in and out of the cell and assists the efflux of glucose following gluconeogenesis [11]. GLUT2 levels have been shown to be increased in insulin-deficient diabetes [12], obesity, and insulin resistance, which exacerbates metabolic dysfunction in non-alcoholic fatty liver disease [13]. GLUT1, as a hepatic influx transporter of glucose, is expressed mainly on the sinusoidal membrane of hepatocytes [14]. High doses of glucose as reported in a streptozotocin diabetes mellitus model were shown to dysregulate GLUT1 protein expression or localization and demonstrated to be normalized by metformin [15]. Moreover, chronic hyperglycemia has been demonstrated to down-regulate GLUT1 and GLUT3 expression in the rat brain, which might be an adaptive reaction of the body to inhibit internalization of excessive glucose by these cells, leading to cell damage [16]. Decreased levels of GLUT4 have also been reported in a mouse obesity model [17]. Although there are not many studies regarding the role of GLUT3 and GLUT4 in liver, their hepatic expression has been reported. Moreover, it has been shown that GLUT3 translocates to the plasma membrane in response to insulin, and insulin-stimulated glucose uptake was abolished with siRNA-mediated GLUT3 knockdown [18].

Transthyretin (TTR), a 55 kDa evolutionarily conserved protein, mainly produced by the liver and by the choroid plexus and secreted into the blood and cerebrospinal fluid (CSF), respectively, functions as a transporter of thyroxin and retinol [19]. In the acute inflammatory states, cytokines decrease TTR and retinol-binding protein (RBP) concentrations [20], and thus TTR is considered a negative acute-phase protein [21]. Recently, the role of TTR in energy and glucose metabolism has attracted the attention of researchers and been investigated in different pathological conditions.

It is important to note that TTR levels are increased in type 2 diabetes [22], while they are decreased in type 1 diabetes [23], suggesting that TTR levels must be kept in balance within healthy intervals, not high nor low, otherwise they may contribute to the development of pathological conditions. Additionally, studies are sometimes contradictory; for instance, in a study, TTR knock-out (KO) mice showed no difference either in body weight and white adipose tissue morphology or in basal or fasting-induced circulating levels of glucose, lipids, and leptin [24]. On the other hand, in a more recent study, it was demonstrated that TTR KO mice showed impaired recovery of blood glucose and glucagon levels during the insulin tolerance test [25]. In the absence of TTR, lower levels of glucagon were significantly induced in the islets of Langerhans, suggesting that TTR may have important roles in glucose homeostasis by regulating the expression of glucagon [25]. Furthermore, TTR has been shown to promote pancreatic insulin release, protect against β-cell death [26], bind to glucose-regulated proteins [27], and increase expression of glycolytic enzymes such as pyruvate kinase M type (PKM) in astrocytes, mediated by the cAMP/PKA-dependent pathway [28]. Further, TTR has been shown to be internalized by the pancreatic [27], hepatic, and kidney cells [29]. Moreover, expression of TTR seems to be altered in endocrine cells of the Langerhans islet in type 2 diabetes [30]. In a recent study, authors suggest that TTR, which is elevated in insulin-resistant ob/ob (obese) mice [22] and in some insulin-resistant humans [31], is a key determinant by regulating the levels of RBP4, since RBP4 binding to TTR reduces the glomerular filtration rate of RBP4 and retains it in the blood [32]; Elevated levels of RBP4 cause insulin resistance and impair insulin signaling in mice. In the referred work, the use of TTR-antisense oligonucleotides (ASO) decreased LDL cholesterol in high-fat-diet-fed mice, inhibited hepatic glucose production, elevated glucose uptake into skeletal and cardiac muscle, and further increased insulin signaling in muscle [33].

In this work, we aimed at assessing the involvement of TTR in glucose homeodynamics in both plasma and liver cells and at investigating the underlying mechanism, namely the impact of TTR on the expression of glucose transporters, expression of PKM, and production of the glucose-derived metabolites, all involved in the glucose metabolism. Finally, we aimed at evaluating the role of TTR in mitochondrial function.

## 2. Results

### 2.1. TTR Haploinsufficiency Increases Glucose Levels in Plasma and in Extracellular Culture Media of Hepatocytes

**Figure 1:**
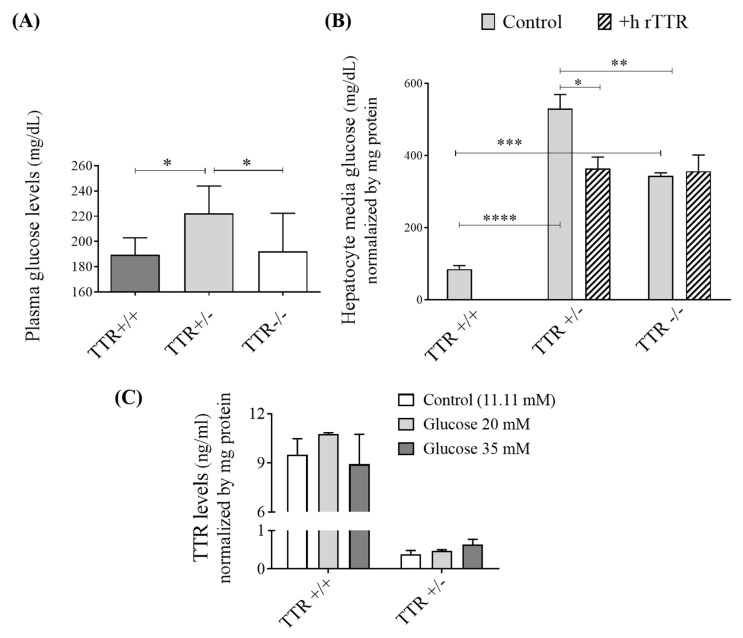
Effect of TTR and glucose on each other. (**A**) In mice, plasma glucose levels were shown to be higher in TTR+/− mice (*N* = 11), while TTR−/− (*N* = 7) showed no alteration as compared to plasmas from TTR+/+ animals (*N* = 5). (**B**) In primary hepatocytes, glucose levels, normalized by mg protein, were also higher in the extracellular culture media of primary hepatocytes derived from TTR+/− and TTR−/− mice. Further addition of h rTTR to the media of TTR+/− hepatocytes resulted in decreased glucose levels, while it did not alter glucose concentration in hepatocytes from TTR−/− mice. (**C**) In primary hepatocytes, different concentrations of glucose did not produce any alteration in the concentration of secreted TTR (*N* = 3 for each condition). Data are expressed as mean ± SD. Significant results are indicated by * when *p* < 0.05, ** when *p* < 0.01, *** when *p* < 0.001 and **** when *p* < 0.0001.

To study the role of TTR on glucose metabolism, we firstly evaluated glucose levels under fasting conditions, to guarantee reliable results, in plasmas of mice with different TTR backgrounds. As presented in Figure 1A, mice with TTR haploinsufficiency (TTR+/−) showed higher plasma glucose levels (222.19 ± 11.63 mg/dL) compared to those from TTR+/+ animals (194.82 ± 13.43 mg/dL). However, TTR deficiency in TTR−/− mice did not induce any alteration in glucose (192.16 ± 11.10 mg/dL), confirming previous reports [24], which may be explained by compensation mechanisms developed to overcome the total absence of TTR at all stages of development of these animals.

Then, we evaluated the glucose levels in the extracellular culture media of primary hepatocytes (Figure 1B). For these studies, we used non-fasting animals, because the cells were manipulated and cultivated for at least 48 h under controlled media conditions (with 11.11 mM glucose). Then, the media was changed 24 h before media collection for analyses. As such, these conditions mimic fasting.

The results confirmed the observations in the plasmas, as TTR+/− hepatocytes presented the highest glucose levels in their media (530.32 ± 67.81 mg/dL per mg protein). The increased glucose concentration in the media suggests that the hepatocytes could be central to the increased plasma glucose level seen in TTR+/− mice either by decreased uptake or increased efflux. Importantly, the addition of h rTTR in the media of TTR+/− hepatocytes partially rescued the phenotype and significantly decreased the glucose levels (364.57 ± 55.18 mg/dL per mg protein). We also observed that glucose in the extracellular culture media of hepatocytes isolated from TTR−/− mice was significantly higher than in the TTR+/+ (respectively, 344.21 ± 14.44 versus 84.77 ± 17.20 mg/dL per mg protein), differently from the measurements in plasmas, but still not as high as in the TTR+/− media. Additionally, the addition of h rTTR did not alter glucose concentration in hepatocytes from TTR−/− mice (356.84 ± 78.11 mg/dL per mg protein), which once again may indicate that these mice developed other compensation mechanisms.

We then questioned if glucose can also influence TTR levels or not. In order to investigate a possible modulation of TTR expression by glucose, we incubated primary hepatocytes with different concentrations of glucose, and as can be observed in Figure 1C, TTR protein levels were not altered in these conditions. Thus, our results indicate that TTR regulates glucose levels and not the other way around.

### 2.2. TTR Haploinsufficiency Alters the Expression of Glucose Transporters

The higher levels of glucose observed in plasmas and in the extracellular culture media of TTR+/− mice can be explained by altered glucose transport, decreased glucose catabolism, and/or increased glucose anabolism. A previous study demonstrated that TTR binds to glucose-regulated proteins [27], and glucose transporters are the main family mediating glucose uptake in tissues. Thus, we then studied the impact of TTR on GLUT transporters, at the levels of transcript and of protein, in mouse liver and hepatocytes, respectively, and also in the HepG2 cell line.

**Figure 2:**
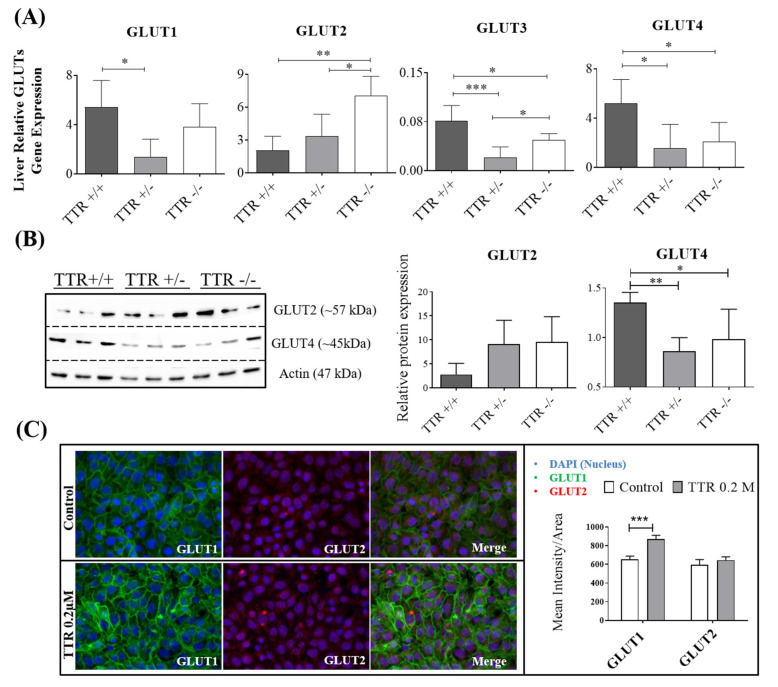
Effect of TTR on hepatic glucose transporters, assessed by qRT-PCR, Western blot, and immunocytochemistry. (**A**) In mice, at mRNA level, haploinsufficiency of TTR in TTR+/− mice livers significantly decreased expression of GLUT1, GLUT3, and GLUT4, compared to TTR+/+ livers, while TTR deficiency in TTR−/− mice livers showed lower expression of GLUT3 and GLUT4 and higher expression of GLUT2, compared to TTR+/+ livers (*N* = 4 for each genotype). (**B**) In primary hepatocytes, and at protein levels, cells derived from TTR+/− and TTR−/− mice showed lower expression of GLUT4 (*N* = 3 for each genotype). (**C**) HepG2 cells also showed higher protein levels of GLUT1 when incubated with h rTTR (0.2 µM), compared to untreated cells (*N* = 4 for each condition). Data are expressed as mean ± SD. Significant results are indicated by * when *p* < 0.05, ** when *p* < 0.01, and *** when *p* < 0.001.

For GLUT1, GLUT3, and GLUT4, transcript levels (Figure 2A) were observed to be decreased in TTR+/− mice livers, explaining the previous results and indicating that TTR haploinsufficiency inhibits the expression of glucose transporters, resulting in decreased glucose uptake by these cells. TTR−/− mice livers also showed significantly reduced levels of GLUT3 and GLUT4 gene transcripts compared to TTR+/+ mice, whereas GLUT1 decrease was not statistically significant. For GLUT2, we observed increased mRNA levels in the livers of TTR−/− compared to both TTR+/+ and TTR+/− mice. At protein level (Figure 2B), we could only evaluate GLUT4 and GLUT2 levels in primary hepatocytes and observed the same behavior as the transcripts; GLUT4 was shown to be expressed in lower levels in TTR+/− and TTR−/− primary hepatocytes. For GLUT2, we could not observe significant changes in any of the genotypes, although this transporter tended to be increased when compared to TTR+/+, with the corresponding median (interquartile range; IQR) levels being 1.85 (1.63–4.38) for TTR+/+, while 11.05 (3.81–13.34) and *p* value = 0.1, and 6.89 (5.33–15.14) and *p* value = 0.08, respectively, for TTR+/− and TTR−/− animals. As a previous study indicated, GLUT2 expression is dependent on glucose levels [34]; thus, its increased expression in TTR−/− and in TTR+/− might be a response to the higher glucose observed in the extracellular culture media of primary hepatocytes derived from these mice.

To investigate if the observed effects are induced directly by TTR, HepG2 cells were incubated with or without h rTTR, and GLUT1 and GLUT2 protein expression were assessed using immunocytochemistry. As shown in Figure 2C, GLUT1 levels were increased in the presence of 0.2 µM TTR, whereas GLUT2 levels were not altered at this concentration of h rTTR.

### 2.3. TTR Haploinsufficiency Leads to a Reduction in Expression of Glycolytic Enzyme, PKM, but Not in Production of Acetate, Lactate, or Alanine

**Figure 3:**
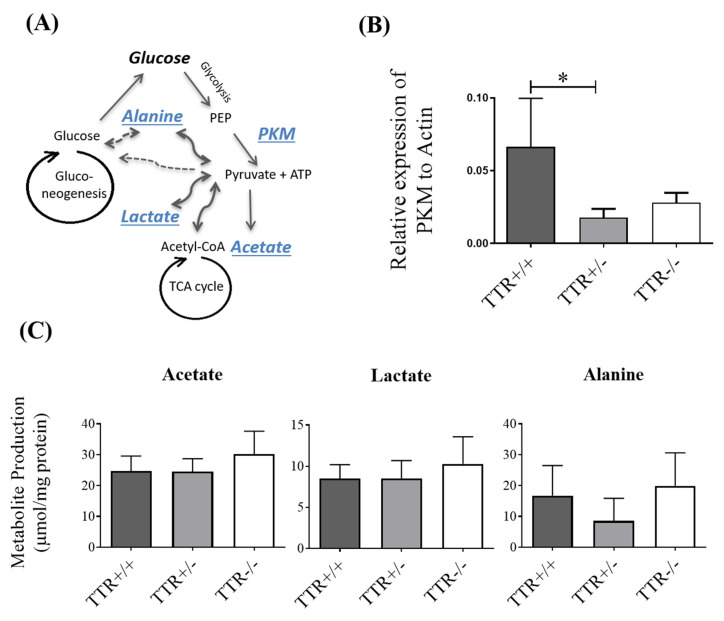
Effect of TTR on the expression of liver PKM enzyme and hepatic production of glucose-derived metabolites. (**A**) A simple schematic representation of the metabolic pathways of glucose: during glycolysis, glucose is converted to pyruvate, through the action of PKM on PEP, producing ATP. Pyruvate can further turn into acetate, lactate, and alanine and/or enter the mitochondrial TCA cycle for further production of energy. Glucose can also be produced through gluconeogenesis in the liver. (**B**) In mouse livers, the relative expression of PKM, assessed by qRT-PCR, was shown to be lower in TTR+/− livers compared to those from TTR+/+ animals (*N* = 4 for each genotype). (**C**) In primary hepatocytes, the production of acetate, lactate, and alanine was assessed by ^1^H-NMR spectroscopy. No significant differences were observed in their production by hepatocytes from different TTR backgrounds mice (*N* = 6 for each condition). Data are expressed as mean ± SD. Significant results are indicated by * when *p* < 0.05.

In order to have an integrative view regarding the effect of TTR on glucose metabolism and the fate of consumed glucose in the liver (Figure 3A), we then evaluated the expression of the PKM enzyme, responsible for converting phosphoenolpyruvate (PEP) to pyruvate during glycolysis. As shown in Figure 3B, the transcript levels of PKM were decreased in mice with TTR haploinsufficiency (Median (IQR) levels were 0.027 (0.016–0.041) for TTR−/−, 0.012 (0.011–0.030) for TTR +/−, and 0.0636 (0.0362–0.0996) for TTR+/+ animals). As studies show that glucose can stimulate transcription of the *PKM* gene [35] and that hyperglycemia and diabetes decrease PKM activity in mouse glomeruli [36], it suggests that PKM activation increases glucose metabolic flux, and that our observation of decreased PKM expression in TTR+/− livers could suggest a decreased utilization of glucose for glycolysis, leading to less ATP being produced in this step of glycolysis.

We then evaluated the levels of glucose-derived metabolites, namely lactate, acetate, and alanine, in the culture medium of primary hepatocytes derived from mice with different TTR backgrounds, and as shown in Figure 3C, there were no differences in the production of acetate, lactate, and alanine between hepatocytes derived from mice with two copies (24.79 ± 4.77, 8.50 ± 1.69, and 16.74 ± 9.78 μmol/mg protein, respectively), one copy (24.54 ± 4.15, 8.54 ± 2.14, and 8.55 ± 7.83 μmol/mg protein, respectively) or no copies (30.23 ± 7.40, 10.29 ± 3.29, and 19.94 ± 10.64 μmol/mg protein, respectively) of the *TTR* gene. Thus, we speculate that the higher glucose levels in TTR+/− animals (Figure 1A,B), together with the observation of unchanged production of the metabolites, might underlie TCA cycle dysfunction, impaired mitochondrial activity, and/or oxidative damage.

### 2.4. TTR Affects Hepatic Mitochondrial Density and Expression of Total OXPHOS

**Figure 4:**
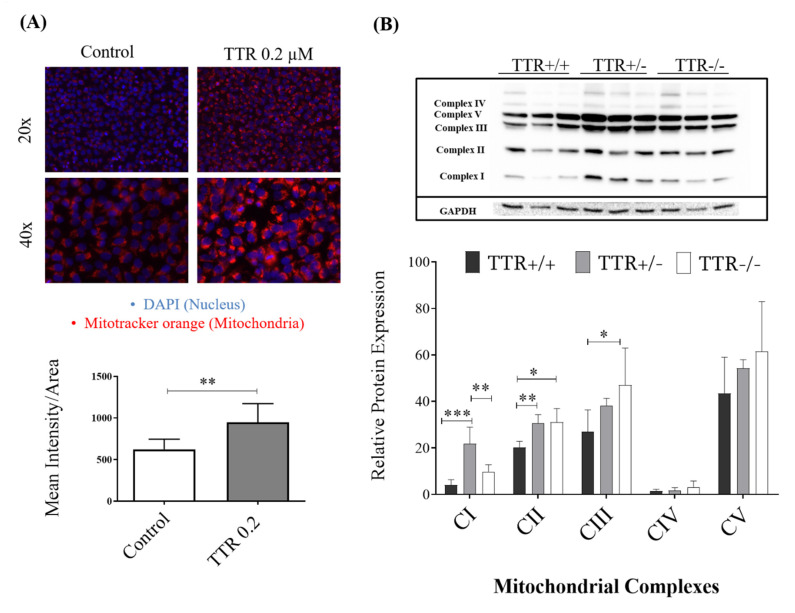
Effect of TTR on mitochondria as assessed by fluorescent microscopy and Western blot. (**A**) HepG2 cells treated with h rTTR showed significantly higher mitochondrial density compared to untreated ones (*N* = 5 for each condition). (**B**) In mouse liver, and as assessed by Western blot, higher protein levels of mitochondrial CI and CII were observed in TTR+/− mice, and higher CII and CIII in TTR−/− mice, compared to TTR+/+ animals (*N* = 3 for each genotype). Data are expressed as mean ± SD. Significant results are indicated by * when *p* < 0.05, ** when *p* < 0.01, and *** when *p* < 0.001.

To study the influence of TTR on mitochondrial function, we assessed levels of mitochondrial staining using immunofluorescence. As shown in Figure 4A, there was a significant increase in the probe fluorescence intensity in the presence of h rTTR, indicating the higher mitochondrial density in these cells.

We also studied protein expression levels of mitochondrial OXPHOS complexes (CI, CII, CIII, CIV, and CV) in liver protein extracts from mice with different TTR backgrounds. As shown in Figure 4B, we observed higher expression of mitochondrial complexes CI and CII in livers of TTR+/− compared to TTR+/+ animals. In TTR−/− mice, CII and CIII complexes were higher when compared to TTR+/+ animals.

## 3. Discussion

TTR, formerly called prealbumin due to its electrophoretic characteristics, is a plasma and CSF protein, produced and secreted mainly by the liver and choroid plexus and to a lesser extent by the retinal pigment epithelium and the islets of Langerhans.

Although the involvement of TTR in the transport of thyroid hormones [37] and retinol through binding to RBP [38], as well as in the development of familial amyloidotic polyneuropathy (FAP) [39], is well established, its other roles, such as neuroprotective and metabolic roles, began to be explored only recently.

There is a large range for “normal” serum TTR levels, i.e., ∼300–330 mg/L in males and ∼250–270 mg/L in females [20]; however, lower and higher TTR levels seem to be cause or consequence of many pathological conditions. For instance, it has been long known that TTR levels are decreased in malnutrition, inflammation [20], and ovarian cancer [40]. Moreover, TTR stability and/or its levels are reduced in type 1 diabetes [23,41] and in Alzheimer’s Disease [42,43,44]. Further, low serum TTR levels were shown to be associated with an increased risk of mortality in patients with dialysis [45] and acute ischemic stroke [46]. On the other hand, TTR levels have been demonstrated to be increased in the mouse model of obesity [47], type 2 diabetes [22,33], and in pregnant women with gestational diabetes [48,49].

To deepen the current knowledge on the role of TTR in glucose metabolism, we firstly analyzed the levels of glucose in mouse plasmas and in the extracellular culture media from primary hepatocytes derived from mice with different TTR genetic backgrounds. We observed that in fasting mice with insufficient TTR (TTR+/−), plasma glucose was increased compared to TTR+/+ and TTR−/− animals. Interestingly, in another study, it has also been shown that there were no differences either in basal or fasting-induced circulating levels of plasma glucose between TTR+/+ and TTR−/− mice [24]. However, in the referred study, TTR+/− animals were not investigated. Previous works on Alzheimer’s Disease/TTR (AD/TTR) mice models have also shown that the TTR-KO animals did not always follow the expected trend compared to the heterozygous models [50,51]. Some of the authors hypothesized that AD mice bearing the homozygous deletion of the *TTR* gene might undergo transcriptional remodelling through which compensatory mechanisms may occur, overcoming the deleterious effects of the total lack of TTR in the pathogenesis of AD [50]. It is known that cells have redundant mechanisms, and it is accepted that compensation mechanisms can be activated to overcome, in this case, the total absence of TTR, occurring at all stages of development of the animals. Moreover, our results from glucose levels in hepatocytes media confirmed what was observed in the plasmas, as media of TTR+/− hepatocytes contained the highest glucose levels. Additionally, there was a significant increase in glucose levels in media of TTR−/− hepatocytes compared to those from TTR+/+ mice, but still lower than those derived from TTR+/− animals. This difference, as compared to the glucose measured in plasma from TTR−/− animals, can be explained by the differences between the two models (plasmas versus cultured hepatocytes) and/or by the impact that other factors and organs, such as the pancreas, can have in the plasma glucose, which are not all contemplated in the cellular model. In the future, it will be important to investigate the glucose metabolism in TTR conditional animal models, where the expression of TTR exists before birth and is only blocked or reduced at a certain age, providing a closer model to the events occurring in certain human pathologies. Studies aiming to establish correlations between TTR and glucose blood levels, both in patients with diabetes and in healthy controls, can also shed light on this topic.

We also investigated the expression of glucose transporters as a possible pathway affected by TTR and found that GLUT3 and GLUT4 were decreased in both TTR+/− and TTR−/−, while GLUT1 was reduced only in TTR+/− mice. As for GLUT2, this glucose transporter was increased in TTR−/− animals when analyzing the gene transcript, while the protein assessment only showed a non-significant increasing trend, both in TTR+/− (*p* value = 0.1) and in TTR−/− mice (*p* value = 0.08). *Glut2* is a glucose-sensitive gene in liver cells [52], and previous studies showed that GLUT2 mRNA expression is increased in streptozocin-induced diabetes [12] and is dependent on glucose levels [34,53]. As GLUT2 is a bidirectional transporter for glucose, this result may suggest two ideas: Firstly, as an influx transporter, the observed increase can be a compensation mechanism to increase the transport of glucose into the cells, resulting in what was observed in TTR−/− plasmas (Figure 1A). Secondly, as an efflux transporter, this increase can suggest higher production and/or efflux of glucose out to the plasma. Adding this to the decrease in glucose consumption originated from the decrease in other glucose transporters, the diabetic condition is therefore exacerbated in mice with TTR haploinsufficiency and deficiency compared to those having the normal levels of TTR. Yet, other compensation mechanisms need to be activated in TTR−/− animals to justify their lower glucose levels in plasma/hepatic media when compared to those from TTR+/− mice, which, as already discussed, might be the case of an effect of TTR on insulin and its related receptors, glucose production, and glycogen content, in TTR deficiency versus TTR haploinsufficiency mice models. Importantly, the investigation of the consequences of TTR haploinsufficiency, mimicked in TTR+/− mice, is probably more important and relevant than to study the effects of the total absence of TTR (as in TTR−/− mice), since there are no reports of complete absence of TTR in humans.

Once inside the cells, glucose is mostly metabolized to pyruvate and produces ATP in the process of glycolysis. In fact, pyruvate is at a crossroads of several metabolic pathways; it can be converted to alanine by alanine aminotransferase or it can originate lactate through the action of lactate dehydrogenase. Nevertheless, the majority of pyruvate is further metabolized to acetyl-CoA entering the TCA cycle in the mitochondria [54]. Therefore, to further explore pathways that can be involved in the TTR–glucose relation and to study other reasons/consequences of increased levels of glucose in TTR+/− animals, we then evaluated the liver expression of the PKM enzyme, responsible for converting PEP to pyruvate in the last step of glycolysis, and also analyzed the levels of glucose-derived metabolites, namely lactate, acetate, and alanine in extracellular culture media of primary hepatocytes. We observed that insufficient TTR leads to decreased expression of PKM, which also results in less ATP being produced in this step of glycolysis. We did not observe any significant alteration in production of the aforementioned metabolites, suggesting no alteration in these metabolic pathways and that TTR may influence the downstream pathways and mechanisms, namely mitochondrial activity and/or oxidative damage.

Thus, finally, we evaluated the effect of TTR on mitochondrial function and its oxidative phosphorylation and observed significant increase in protein expression of complex I and II of the electron transport chain in TTR haploinsufficiency and of complex II and III in TTR deficiency. Interestingly, it has been shown that conditions that decline GLUT1-mediated glucose transport result in increased ROS levels in myoblasts [55], similar to our observation in livers. As ROS have been shown to decrease mitochondrial density and increase leakage of proton into the mitochondria, resulting in decreased mitochondrial membrane potential [56], we also studied the effect of TTR on mitochondrial density using MitoTracker^®^ in HepG2 cells. TTR was shown to increase mitochondrial density, and thus its insufficiency may negatively affect mitochondrial activity. In other studies, it has also been shown that exposing HepG2 cells to high amounts of glucose decreases the mitochondrial DNA content and inhibits mitochondrial function [57,58]. In a recent study, AD-model neurons demonstrated both decreased expression of TTR and increased mitochondrial dysfunction [59]. They also showed that in 3xTg-AD mice, a triple-transgenic mouse model of AD with decreased mitochondrial respiration, induction of human mitochondrial transcriptional factor A (TFAM) expression, known to protect mitochondria from oxidative stress, significantly improved cognitive function and elevated expression of TTR. Furthermore, the authors demonstrated a direct interaction between TFAM and TTR in neurons, using immunoprecipitation [59], suggesting an important role for TTR in mitochondrial function. Future work should also address mitochondrial oxidative stress, such as the levels of ROS and lipid peroxidation, in models with different TTR levels.

**Figure 5:**
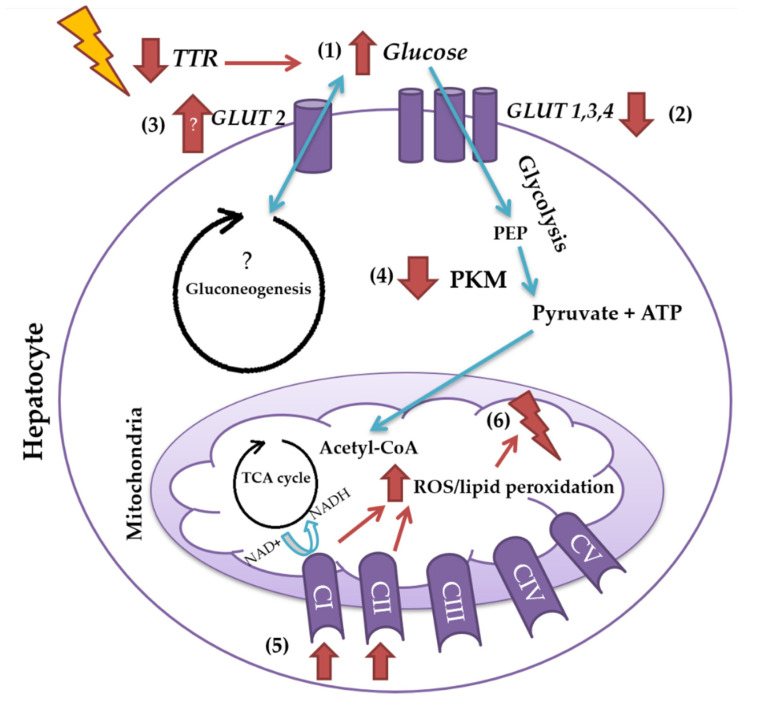
Graphical summary of the results obtained in the present study, illustrating the effects of TTR insufficiency on glucose metabolism of the liver. Insufficiency of TTR, which can result from its tetrameric instability and consequent increased clearance, can increase extra-hepatic glucose levels (1), which can be, at least partially, due to its negative effect on the expression of influx glucose transporters, GLUT1, GLUT3, and GLUT4 (2) and increased expression of efflux glucose transporter, GLUT2 (3). Moreover, the decrease in the expression of glycolytic enzyme, PKM (4), and the increase in mitochondrial CI and CII of the electron transport chain (5) are observed in the TTR haploinsufficiency mouse model, which in turn leads to higher levels of ROS and/or lipid peroxidation and ends up compromising the mitochondrial density and function (6).

Altogether, our results showed for the first time that TTR insufficiency, which can result from its tetrameric instability, as described in familial amyloid polyneuropathy [60], in Alzheimer’s disease [42,44] and in diabetes type I [41], can cause hepatic metabolic dysfunction, as deduced by the higher extracellular or plasma glucose levels, lower expression of influx glucose transporters, lower expression of glycolytic enzyme PKM, a higher degree of oxidative phosphorylation, and thus dysregulation in mitochondrial function (see Figure 5 for a possible interpretation of our results). Our results highlighted a physiological role for TTR in hepatic glucose metabolism and may provide relevant information for the design of TTR-based therapeutic strategies for diabetes and other disorders in which glucose metabolism is impaired.

## 4. Materials and Methods

### 4.1. TTR Production

Human recombinant TTR (h rTTR) was produced in a bacterial expression system using *Escherichia coli* BL21 and purified as previously described [61]. Briefly, after growing the bacteria, the protein was isolated and purified by preparative gel electrophoresis after ion-exchange chromatography. Protein concentration was determined by the Bradford method (Bio-Rad, Algés, Portugal), using bovine serum albumin (BSA) as standard.

### 4.2. Animals

In this work, male and female TTR wild type mice (TTR+/+), TTR-heterozygous mice (TTR+/−; as TTR haploinsufficiency model), and TTR KO mice (TTR−/−; as TTR deficiency model), in an SV129 background [62], aged 2–3 months, were randomly selected and used in the studies described below. Animals were housed in a controlled environment (12-h light/dark cycle; temperature 22 ± 2 °C; humidity 45–65%), with freely available food (2014S da teklad (Envigo, Barcelona, Spain)) and water.

All efforts were made to minimize pain and distress. All procedures involving animals were approved by the Institute for Research and Innovation in Health Sciences (i3S) Animal Ethics Committee (14 March 2014) and in agreement with the animal ethics regulation from Directive 2010/63/EU.

### 4.3. Plasma and Organ Collection

To collect plasma for glucose measurement, mice previously fasted from food for 7 h. Mice were then profoundly anesthetized with an anesthetic combination of ketamine (75 mg/Kg) and medetomidine (1 mg/Kg) by intraperitoneal injection. Blood was collected from the inferior vena cava with syringes with EDTA, followed by centrifugation at 950 g for 10 min (min) at room temperature (RT). Supernatants were collected and frozen at −80 °C until used.

To collect mice livers, non-fasted animals were perfused with PBS through the vena cava for 20 min. Livers were then collected and stored frozen at −80 °C until used.

### 4.4. Primary Hepatocytes and HepG2 Cells Culture

Primary hepatocytes derived from mice with different TTR backgrounds were obtained by two-step collagenase perfusion of livers, as previously described [63,64]. Briefly, a cannula was inserted into the portal vein, and a perfusion medium (HBSS 1x medium containing 0.025 M HEPES and 2mM EDTA) was allowed to perfuse through the liver (5 mL/min). Then, the vena cava was cut immediately. After 10 min, perfusion medium was substituted by collagenase solution (Williams E medium (WE, Gibco™, Carlsbad, CA, USA) containing 10% FBS, 3 mM CaCl_2_, 0.01 M HEPES, and 0.25 mg/mL Collagenase type V (Sigma-Aldrich, Sintra, Portugal) for another 10 min. The entire perfused liver was then collected in an isolation medium (WE medium containing 10% FBS, 2 mM EDTA, and 0.01 M HEPES), followed by centrifugations, counting live cells, and finally seeding with an attachment medium (WE medium containing 10% FBS and 0.01 M HEPES) in 25 cm^2^ flasks or 6-well plates for 3 h. Then, the medium was changed to a stimulation medium (WE medium containing 2× penicillin-streptomycin, 0.01 M HEPES, 0.04% Fungizone, 0.05 mM Dexamethasone, 1 μM Insulin, and 0.05 mM 2 Mercapto-ethanol), and after 48 h, the experiments were performed in stimulation media.

HepG2 cells, a human hepatoma line, was grown in Dulbecco’s modified Eagle medium (DMEM) (Lonza, Verviers, Belgium) and Ham’s F-12 media (Lonza, Verviers, Belgium), supplemented with 10% FBS (Gibco™, Carlsbad, CA, USA), 100 U/mL penicillin–streptomycin (Gibco™, Carlsbad, CA, USA), 2 mM L-glutamine (Gibco™), and 1X nonessential amino acid solution (Lonza, Verviers, Belgium).

### 4.5. Protein Extraction

Livers and primary hepatocytes obtained from mice with different TTR backgrounds were homogenized in lysis buffer (20 mM MOPS pH 7.0; 2 mM EGTA; 5 mM EDTA; 30 mM sodium fluoride; 60 mM β -glycerophosphate pH 7.2; 20 mM sodium pyrophosphate; 1 mM sodium orthovanadate; 1% triton X-100), 1 mM phenylmethylsulphonyl fluoride (PMSF) and protease inhibitors (GE healthcare, Chicago, IL, USA), followed by 20 min incubation on ice. Extracts were then centrifuged at 18,600× *g* for 20 min at 4 °C, and supernatants were used for protein analysis. The total protein concentration of the extracts was quantified by the Bradford method (Bio-Rad, Hercules, CA, USA), using BSA as standard.

### 4.6. Glucose Measurement in Mouse Plasma and Extracellular Culture Media of Primary Hepatocytes

Glucose levels in plasmas (mg/dL) from fasted mice and in the extracellular culture medium of primary hepatocytes previously incubated for 24 h, with or without additional exogenous h rTTR (0.2 µM), were measured using a glucose assay kit (Abcam, Cambridge, UK) following the manufacturer’s instructions. In the case of hepatocytes, proteins from cells were also extracted, quantified, and used to normalize the levels of glucose in the supernatants, and finally, the results were expressed as glucose levels (mg/dL) per mg of protein.

### 4.7. Mouse TTR ELISA

Mouse TTR in extracellular culture media of primary hepatocytes incubated with different concentrations of glucose, i.e., 11.11 mM (control culture media), 20 mM, and 35 mM, was quantified using the Mouse Prealbumin ELISA Kit (MyBioSource, San Diego, CA, USA) according to the manufacturer’s instructions. Data are expressed in ng/L per mg of protein.

### 4.8. Real-Time PCR

Liver RNA was isolated using Trizol (Alfagene^®^, Carcavelos, Portugal). For the reverse transcription to cDNA, 4 μg of RNA was applied using the First-Strand cDNA Synthesis Kit (NZYtech, Lisbon, Portugal). The reaction mix was then subjected to quantitative real-time PCR with the SYBR Green reporter (iQ SYBR Green supermix, BioRad) to detect expression levels of each gene of interest and, as reference genes, glyceraldehyde 3-phosphate dehydrogenase (GAPDH) or β-Actin. The primers used in this project are presented in Table 1. Reactions were run in a Bio-Rad iCycler. The relative levels of expression were quantified and analyzed by Bio-Rad iQ5 software. Data were calculated using the ΔCT Method using a reference gene before statistical analysis was performed.

### 4.9. Immunofluorescence for GLUTs

HepG2 cells, grown on round glass coverslips, pre-coated with rat tail collagen type I solution, were incubated with or without h rTTR (0.2 µM) overnight, and then washed with PBS, fixed with acetone for 7 min at −20 °C and blocked with 5% BSA, followed by incubation with primary antibodies mouse anti-human GLUT1 (Abcam, Cambridge, 3:100) and rabbit anti-human GLUT2 (Abcam, Cambridge, 1:100). After washing, cells were incubated with anti-mouse Alexa Fluor-488 and anti-rabbit Alexa Fluor-568 IgG antibodies (Invitrogen, 1:2000), respectively. Then, coverslips were mounted with Fluoroshield™ with DAPI (Sigma-Aldrich, Sintra, Portugal) and visualized and photographed with Zeiss Axio Imager Z1 equipped with an Axiocam MR3.0 camera and Axiovision 4.7 software. Finally, fluorescence mean intensity per area in the images was quantified using Fiji software [65].

### 4.10. Proton Nuclear Magnetic Resonance (1H NMR) Spectroscopy

To determine the concentration of metabolites in the extracellular culture media of primary hepatocytes, media were collected from cells, derived from mice with different TTR genetic backgrounds, incubated for 9 h, and stored in −20 °C before ^1^H-NMR spectroscopy quantification. Fully relaxed ^1^H NMR spectra of extracellular media were obtained at 14.1 T, 25 °C, using a Bruker Avance 600 MHz spectrometer equipped with a 5 mm QXI probe with a z-gradient (Bruker Biospin, Karlsruhe, Germany) using standard methods [66]. Sodium fumarate was used as an internal reference (6.50 ppm) to quantify the following metabolites (multiplet, ppm): lactate (doublet, 1.33), alanine (doublet, 1.45), and acetate (singlet, 1.9). The relative areas of ^1^H-NMR resonances were quantified using the curve-fitting routine supplied with the NUTSpro NMR spectral analysis program (Acorn, Livermore, CA, USA). The results are expressed as production in absolute values of µmol per mg total protein content of corresponding cells.

### 4.11. Western Blot Analysis

Protein levels of glucose transporters (GLUTs) in mice primary hepatocytes, as well as mitochondrial complexes (MCs) in mice livers, were studied by Western blot (WB) analysis. Protein extract samples (50 μg) were incubated at 37 °C for 15 min and separated using 10% or 15% SDS-PAGE, respectively, for GLUTs and MCs. Proteins were then transferred to polyvinylidene difluoride (PVDF) membrane (WhatmanTM Ge healthcare–Protan BA 83) for MCs, or to nitrocellulose membrane (GE Healthcare Life Sciences, Chalfont St. Giles, U.K.) for GLUTs, using a wet system (Bio-Rad Criterion Blotter). The membranes were blocked for 3 h at RT, with 5% powdered skimmed milk in PBS containing 0.05% Tween-20 (PBS-T). After blocking, membranes were then incubated with the following primary antibodies in 3% powdered skimmed milk in PBS-T: mouse total OXPHOS (Abcam, Cambridge, Ab110413, 1:5000), rabbit GLUT2 (Abcam, Cambridge, ab54460, 1:500), or goat GLUT4 (Santa cruz, sc-1608, 1:1000), and as reference proteins: rabbit GAPDH (Sigma-Aldrich, Sintra, Portugal, G9545, 1:5000) and mouse β-actin (Sigma-Aldrich, Sintra, Portugal, A5441, 1:5000). Then, washed membranes were incubated for 1 h at RT with anti-mouse (The binding Site, Birmingham, UK; 1:2500), anti-rabbit (The binding Site, Birmingham, UK; 1:5000), or anti-goat (1:10,000) immunoglobulins conjugated with horseradish peroxidase. The blots were developed using ClarityTM Western ECL substrate (Bio-Rad). Finally, proteins were detected and visualized using a chemiluminescence detection system (ChemiDoc, Bio-Rad).

### 4.12. MitoTracker^®^ Analysis

To stain mitochondria in HepG2 cells, MitoTracker^®^ Orange CMTMRos (ThermoFisher Scientific, Boston, MA, USA) was used. Cells grown in coverslips were incubated without or with h rTTR (0.2 µM), overnight. Then, cells were washed and incubated with MitoTracker^®^ (100 nM) for 1 h at 37 °C, washed well with PBS, fixed with acetone for 7 min at −20 °C, and finally mounted with DAPI and photographed and analyzed, as described before.

### 4.13. Statistical Analysis

Statistical analyses were carried out using GraphPad Prism 8 software (San Diego, CA, USA). All quantitative data were expressed as mean ± SD. Initially, data normality was assessed based on whether it followed a Gaussian distribution or not. When found to follow a Gaussian distribution, differences among conditions or groups were analyzed by one-way ANOVA with Tukey’s multiple comparisons test (for three groups) or student *t*-test (for comparing two groups). In the cases of non-Gaussian distribution, data in the text were also expressed as median with the according IQR (Interquartile range), further comparisons between three groups were made by non-parametric Kruskal–Wallis test followed by Dunn’s multiple comparisons test, and comparisons between two groups were made by a Mann–Whitney test. *p*-values lower than 0.05 were considered statistically significant.

## Figures and Tables

**Table 1:** Oligonucleotide sequences of the primers used in this study for the amplification of the selected mice transcripts.

Gene Name	NCBI Reference mRNA Sequence	Sense Primer	Anti-Sense Primer
*Glut1*	NM_011400.3	5′-GCTGCTCAGTGTCATCTTCATC-3′	5′-ATCTGCCGACCCTCTTCTTTC-3′
*Glut*2	NM_031197.2	5′-CAGTCACACCAGCATACACAACAC-3′	5′-CCCGAGCCACCCACCAAAG-3′
*Glut*3	NM_011401.4	5′-CGTCCAGCCGCTTCTCATCTCC-3′	5′-TGACCACGCCTGCTCCAATCG-3′
*Glut*4	NM_009204.2	5′-CCAGCCTACGCCACCATAG-3′	5′-TTCCAGCAGCAGCAGAGC-3′
*PKM*	NM_001253883.1	5′-TGAAGGAGATGATTAAGTC-3′	5′-TAGAGAATGGGATCAGAT-3′
*GAPDH*	NM_001289726.1	5′-GCCTTCCGTGTTCCTACC-3′	5′-AGAGTGGGAGTTGCTGTTG-3′
*β-Actin*	NM_007393	5′-TGTCCACCTTCCAGCAGATGT-3′	5′-AGCTCAGTAACAGTCCGCCTAG-3′

## Data Availability

Not applicable.

## Abbreviations

AD	Alzheimer’s Disease
BSA	Bovine serum albumin
FAP	Familial amyloidotic polyneuropathy
GAPDH	Glyceraldehyde 3-phosphate dehydrogenase
GLUT	Glucose transporter
h rTTR	Human recombinant TTR
IQR	Interquartile range
KO	Knock-out
MCs	Mitochondrial complexes
Min	Minutes
OXPHOS	Oxidative phosphorylation
PKM	Pyruvate Kinase M
RBP	Retinol binding protein
RT	Room temperature
ROS	Reactive oxygen species
TCA	Tricarboxylic acid
TTR	Transthyretin
TTR+/+	TTR wild type mice
TTR+/−	TTR heterozygous mice
TTR−/−	TTR KO mice
WB	Western blot
